# Intercompartmental communication in senescence

**DOI:** 10.1002/2211-5463.70236

**Published:** 2026-03-30

**Authors:** Krystyna Mazan‐Mamczarz, Eleanor J. Wind, Jixiang Leng, Myriam Gorospe

**Affiliations:** ^1^ Laboratory of Genetics and Genomics National Institute on Aging (NIA) Intramural Research Program (IRP), National Institutes of Health (NIH) Baltimore MD USA

**Keywords:** endoplasmic reticulum, Golgi, interorganellar communication, organelles, SASP, senescence

## Abstract

Cellular senescence represents a response to sublethal damage, characterized by persistent growth arrest and a robust pro‐inflammatory trait, the senescence‐associated secretory phenotype (SASP). Senescent cells accumulate in the body with age, promoting tissue dysfunction and age‐related disease. In addition to profound reprogramming of gene expression patterns, senescent cells undergo broad remodeling of cellular compartments, including the plasma membrane, nucleus, endoplasmic reticulum (ER), Golgi apparatus, endolysosomal system, mitochondria, biomolecular condensates, and cytoskeleton. These changes alter the intracellular communication networks required for homeostasis. Here, we review how senescence alters (i) vesicular trafficking along secretory, endocytic, and autophagic routes, (ii) interorganelle contact sites such as those among mitochondria, ER, and lysosomes to modulate lipid and calcium exchange, and (iii) diffusion and transport of regulatory signals across the cytosol and membranes. We discuss how the impaired crosstalk among compartments increases ROS, exacerbates proteostatic stress, impairs clearance of damaged components, and activates p53/p21, p16/Rb, cGAS–STING, NF‐κB, and mTOR pathways, enhancing apoptosis resistance and the SASP. Finally, we highlight emerging technologies to study the senescent organelle ‘interactome’ and identify therapeutic vulnerabilities in age‐associated declines and diseases linked to senescence.

**Impact statement**

We synthesize evidence that cellular senescence arises not only from gene expression changes but also from disrupted interorganelle communication. We discuss defects in vesicle trafficking and organelle contact sites that redefine senescence as failure of the organellar interactome, highlighting future mechanistic work and therapeutic opportunities in age‐related disease.

## Graphical abstract

Senescent cells experience structural changes in the plasma membrane, endoplasmic reticulum, mitochondria, lysosomes, nucleus, and cytoskeleton. These alterations disrupt crosstalk among cellular compartments, impairing vesicular trafficking, contact sites, and molecular flow. Consequently, intracellular communication is compromised, disturbing cellular processes and overall homeostasis.

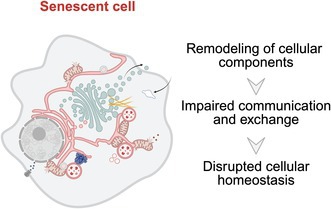

**Abbreviations**
AD: Alzheimer's disease
COPI, COPII: coatomer‐associated protein complexes I and II
ER: endoplasmic reticulum
GRASPs: Golgi reassembly and stacking proteins
LLPS: liquid–liquid phase separation
MAMs: mitochondria‐associated membranes
MCS: membrane contact site
MDVs: mitochondrial‐derived vesicles
MERCs: mitochondria‐ER contact sites
NPC: nuclear pore complex
PD: Parkinson's disease
RNP: ribonucleoprotein
ROS: reactive oxygen species
SASP: senescence‐associated secretory phenotype
SGs: stress granules
TGN: trans‐Golgi network
UPR: unfolded protein response

Cell senescence is a state of indefinite growth arrest resulting from sublethal damage to DNA and other macromolecules. The accumulation of senescent cells in organs with age causes a gradual decline in physiological function and increases age‐related disease. Senescent cells undergo changes in the structure and function of cellular compartments such as the plasma membrane, mitochondria, lysosomes, endoplasmic reticulum (ER), nucleus, and the cytoskeleton. The progressive deterioration of these compartments disrupts intracellular communication systems, such as vesicular trafficking, intercompartmental contact sites, and flow of molecules, impeding proper transfer of information and materials between compartments and across the cell membrane. Disturbance of intracellular communication results in an interruption of coordinated cellular processes that has broad consequences on the function of senescent cells. For instance, defective mitochondria can lead to increased production of reactive oxygen species (ROS), faulty lysosomes can result in heightened β‐galactosidase activity and impaired autophagy, and ER disorganization can compromise the unfolded protein response (UPR) [1]. A malfunctioning crosstalk between organelles in senescent cells triggers positive feedback loops that disrupt crucial regulatory signaling systems, such as the p53/p21, p16/Rb, cGAS‐STING, NF‐κB, and mTOR pathways, contributing to a permanent cell cycle arrest, resistance to cell death, and acquisition of a senescence‐associated secretory phenotype (SASP) whereby cells secrete pro‐inflammatory and matrix remodeling factors [2].

The importance of changes in intracellular communication in aging and age‐related diseases, such as Alzheimer's disease (AD), Parkinson's disease (PD), sarcopenia, osteoporosis, cancer, and cardiovascular disease, is becoming increasingly apparent [3, 4]. However, the effects of disrupted communication between cellular compartments in cellular senescence are not yet fully understood. This review discusses the interactions among cell compartments and how senescence changes these contacts, in turn altering intracellular and extracellular communication.

## Cellular compartmentalization and communication

**Fig. 1:**
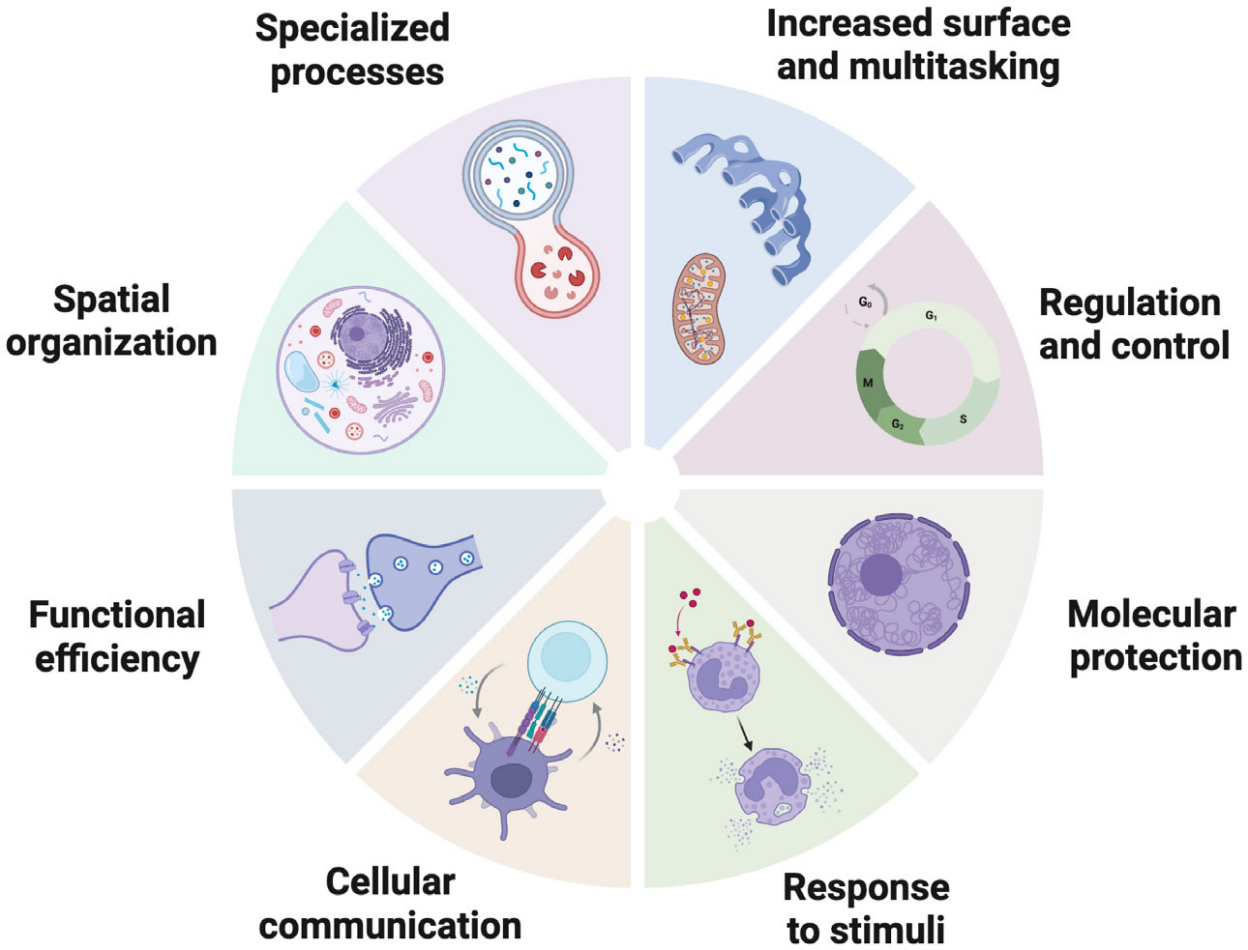
Advantages of subcellular compartmentalization. The compartmental organization of cellular components creates dedicated intracellular environments that facilitate specific biochemical reactions, molecular protection and increased efficiency. Compartmentalization permits specialized functions such as: (i) increasing surface area for protein synthesis and modification, as well as to elicit oxidative phosphorylation to generate ATP; (ii) assembling proteins that function together in complex processes like cell division; (iii) enclosing molecules like DNA to provide protection; (iv) organizing distinct surface areas to respond to stimuli; (v) creating specialized areas to elicit cell‐to‐cell interactions; (vi) coordinating highly specialized domains for extracellular processes like neurotransmission; (vii) walling contents to preserve cell identity; and (viii) building spaces with extreme chemical properties (e.g., pH) to protect other cell components. Image was created using BioRender.

Cellular homeostasis, the stable internal environment essential for proper cell function, is maintained by a complex communication system of cellular compartments coordinated by signaling factors [5, 6, 7, 8]. The internal compartmentalization achieved in eukaryotic cells through membranes or phase separation creates functional units organizing different processes and working together to facilitate the transfer of material and information crucial for cell homeostasis. The compartmentalization of cellular components creates optimal intracellular environments for effectively conducting biochemical reactions, providing molecular protection, and multitasking cellular processes (Fig. 1). It is also essential for coordinating internal communication to precisely and efficiently manage complex cellular activities. Disruption in this communication network leads to abnormal signaling pathways and impaired cell function, in turn triggering cellular states, such as senescence, that exacerbate disease.

### Plasma membrane

The cell membrane (the plasma membrane) plays a crucial role in mediating interactions between the internal and external environments. It supports cell homeostasis through its selective permeability, allowing the passage of specific substances while preventing the movement of others. This precise control over the entry of materials into the cell and the release of cell products directs intracellular processes for energy production, waste disposal, and signaling for a stable internal environment [9]. Physical interaction between the plasma membrane and organelles such as the ER and mitochondria facilitates the function of other cellular compartments [10, 11, 12, 13, 14]. Many of these units are membrane‐enclosed organelles, including the nucleus, ER, Golgi apparatus, endosomes, lysosomes, mitochondria, and peroxisomes; others are membraneless organelles (also called condensates), including stress granules (SGs), nucleoli, and processing bodies (P‐bodies) [15]. While each organelle has a specialized function, they work together in close communication with one another and other cellular components to carry out a wide range of cellular processes [16].

### Nucleus

A fundamental aspect of intracellular communication is the dynamic exchange of information between the nucleus and cytoplasm. As the nucleus contains the cell's genome and carries out transcription of specific genes, a carefully orchestrated export of contents from the nucleus to the cytoplasm is essential for coordinating the transfer of RNAs (protein‐coding mRNAs and regulatory RNAs) to the cytoplasm. This export is facilitated by the nuclear envelope, which separates the nucleus to protect the genetic material, while allowing the exit of molecules to the cytoplasm through nuclear pore complexes [17]. In response to internal and external stimuli, the nucleus regulates organelle function by controlling the production of proteins destined for organelles in anterograde transport, and conversely, organelles send retrograde signals and cytoplasmic content into the nucleus [18]. This nuclear–organellar feedback loop of information exchange coordinates essential cell functions such as protein and lipid biosynthesis, energy production, waste management, and responses to stress conditions.

### Endoplasmic reticulum

The ER is the most widespread organelle within the cell, comprising a continuous membrane‐enclosed interconnected system of tubules and cisternae that extends from the nuclear envelope throughout the cytoplasm to the cell periphery [19]. The extensive ER network serves as a master communication platform in the cell by interconnecting functionally with the nucleus and virtually all cytoplasmic components. It regulates critical cellular processes such as protein synthesis and folding, lipid biosynthesis, carbohydrate metabolism, and calcium homeostasis [20, 21, 22, 23, 24]. The widespread distribution and wide range of functions of the ER make it essential for maintaining cellular balance and serving as a central hub for coordinating the stress response. For example, the excessive accumulation of unfolded or misfolded proteins in the ER disrupts proteostasis and triggers ER stress. In response, the ER sends signals to the nucleus, activating the UPR, which increases the activity of transcription factors and signaling pathways aiming to restore ER homeostasis or induce apoptosis [25, 26].

### Golgi apparatus

The proteins and lipids produced in the ER are transported to the Golgi apparatus for further modification, sorting, and distribution within the cell. The Golgi apparatus, with its stacked and polarized structure, facilitates the processing and movement of molecules from the cis‐Golgi network (entry face near the ER) to the trans‐Golgi network (the Golgi exit), and guide their transport to the final destinations in the cell. The cooperative activity of the ER and the Golgi is fundamental for the function of the cellular secretory pathway, delivering modified molecules to their designated locations within internal cellular components and the extracellular space. An essential aspect of this arrangement is the interaction of the ER and Golgi with endosomes and lysosomes, which are involved in the endocytic pathway to facilitate the processing of materials that have been internalized from outside the cell [27, 28, 29, 30, 31].

### Endolysosomal system

This interconnected vesicular and tubular network, distributed throughout the cytoplasm and coordinated by the ER, is crucial for degrading cellular waste, recycling materials, supplying nutrients, signaling, and regulating plasma membrane proteins [27, 32]. The components of this system communicate closely with other organelles and are regulated by cross‐compartmental mechanisms [33, 34]. This multi‐organelle cooperative activity is exemplified by hydrolytic enzymes, which are synthesized in the ER, modified by the Golgi apparatus, and then transferred into Golgi‐produced vesicles. These vesicles subsequently fuse with late endosomes to deliver the enzymes for degradation in the lysosome [35].

### Mitochondria

Although these organelles are primarily dedicated to energy production, they communicate actively with the ER and other cellular components [36, 37, 38, 39]. Through these interactions, calcium ions are transferred from the ER to mitochondria to regulate a wide range of cellular functions, like energy production, formation of the inflammasome, autophagy, mitophagy, and lipid metabolism. This collaboration is essential for the cell to adapt efficiently to changing conditions [40, 41, 42, 43]; for example, the cooperative fusion of vesicles derived from both the ER and mitochondria is essential for the formation of peroxisomes, key organelles involved in controlling lipid and redox balance within the cell [44, 45].

### Biomolecular condensates

Formed through liquid–liquid phase separation (LLPS), and lacking enclosing membranes, these organelles are composed of specific proteins, nucleic acids, and other molecules that assemble and disassemble in response to cellular cues [46]. The absence of a membrane allows them to quickly exchange materials with the surrounding environment, temporarily store biological macromolecules, and accelerate cellular events in cellular responses. Biomolecular condensates dynamically interplay with other organelles and are regulated by cellular signaling to orchestrate various cellular processes, including ribosome biogenesis, RNA metabolism, stress response, and signal transduction [47, 48, 49]. Examples of membrane‐less cell components include the nucleolus, which facilitates ribosome biogenesis, P‐bodies, which regulate mRNA processing and decay, and SGs, which control the cell's stress response, as recently reviewed [15, 50].

### Cytoskeleton

Intracellular trafficking relies on the cytoskeleton, an internal network of actin microfilaments, intermediate filaments, and microtubules that extends throughout the cell to maintain its shape and enable motility. This system serves as a platform for motor proteins such as myosins, kinesins, and dyneins, which use energy from ATP hydrolysis to move along cytoskeletal filaments and transport molecules, vesicles, and organelles across the cell. The coordinated action of the cytoskeleton and these motor proteins is fundamental for positioning cellular components and regulating cellular functions such as intracellular motility, signal transduction, organelle dynamics, and cell division. The cytoskeleton enables the translocation of organelles like endosomes, lysosomes, and cellular vesicles that regulate major cellular processes such as endocytosis, autophagy, and lipid metabolism [5, 33, 51, 52]. In addition, the function of signaling molecules responding to intracellular and extracellular stimuli, including enzymes (like kinases, GTPases, etc.) and transcription factors, requires their association with the cytoskeleton [51, 53, 54].

## Mechanisms of communication across compartments

**Fig. 2:**
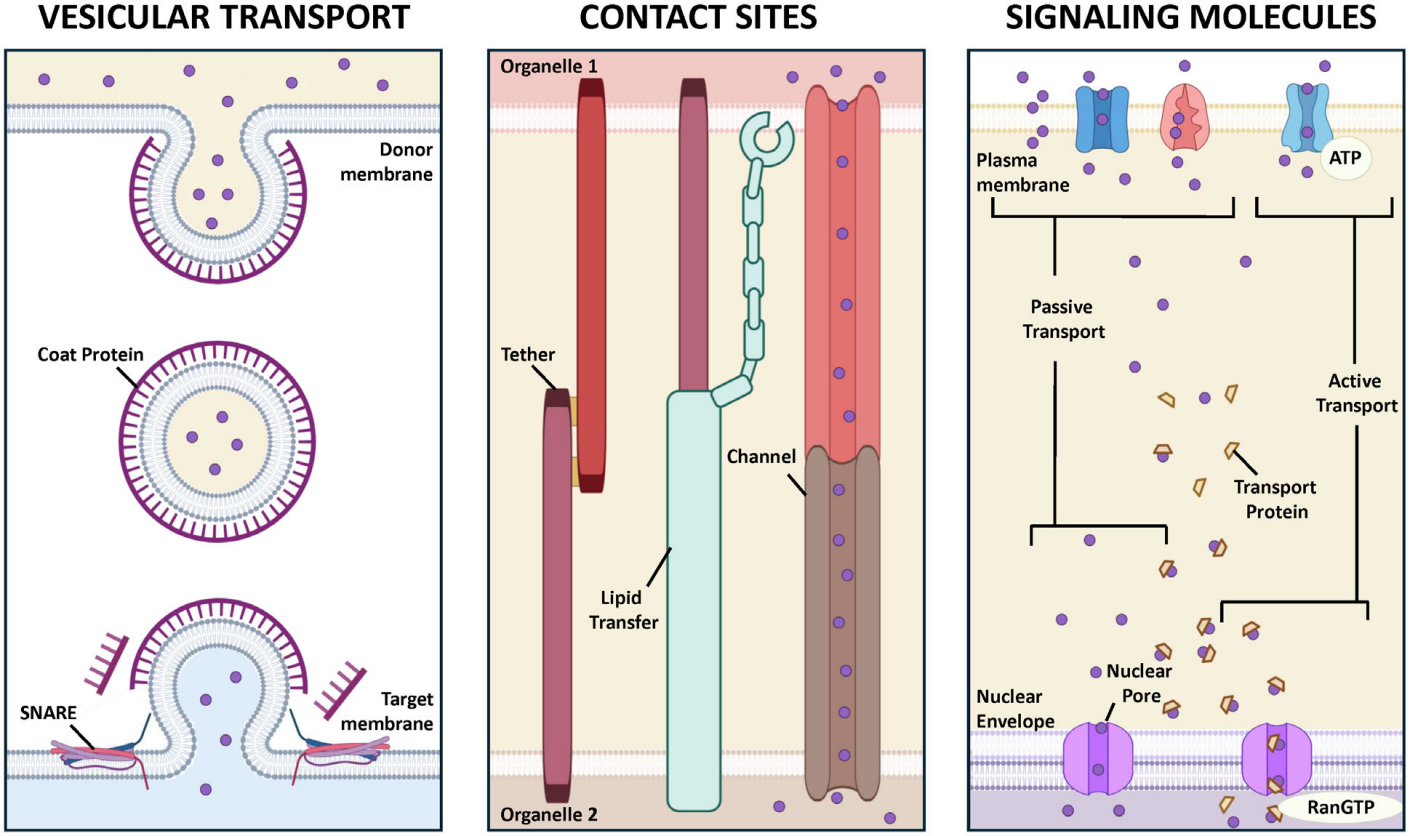
Mechanisms of communication between cell compartments. Schematic illustrating the principal types of communication between cellular compartments, including vesicular trafficking, interorganelle contact sites, and the flow of regulatory signaling molecules. Vesicles transport cargo between donor and target membranes through coat proteins, tethers, and SNAREs. Membrane contact sites permit the direct, nonvesicular transfer of lipids, ions, and metabolites between organelles. Signaling molecules move through passive diffusion or active transport across membranes. Image was created using BioRender.

To maintain activities essential for cell health and respond to intrinsic or extrinsic signals, cells rely on the transfer of molecules both between intracellular compartments and across the cell membrane. The proper function of these processes requires the orchestrated interplay of various cellular components and transport mechanisms. The coordination among cell compartments is mediated by several synchronized mechanisms, including vesicular transport, contact sites, and the flow of regulatory signaling molecules (Fig. 2).

### Intracellular vesicular transport

Vesicular transport is a major mechanism of cellular communication involving small, membrane‐enclosed vesicles that move materials internally within cells and between cells. These vesicles are formed from the donor membrane and are loaded with selected cargo (proteins, lipids, nucleic acids, and other molecules), with the help of specific coat protein systems like coatomer‐associated proteins (COPI, COPII) and clathrin. They are then transported along cytoskeletal filaments by molecular motors and controlled by small GTPases, such as Rab (Ras‐associated binding) proteins, to reach their destination. Proteins in the SNARE (soluble N‐ethylmaleimide‐sensitive factor attachment protein receptor) complex mediate the fusion of the transported vesicles with the membrane of the recipient compartment to deliver their contents [55].

The secretory and endocytic pathways are essential routes for vesicle‐mediated transport and communication. In the secretory pathway, transport vesicles bud off the ER membrane and carry proteins and lipids to the Golgi apparatus for additional processing and sorting. After leaving the Golgi at the trans‐Golgi network (TGN), the materials are enclosed in secretory vesicles and moved to their final destination for secretion, either within the cell or at the plasma membrane for release outside the cell through exocytosis [56]. In the endocytic pathway, molecules from outside the cell are internalized by the plasma membrane and bud off inside the cell to form endocytic vesicles. These vesicles then fuse with early endosomes to sort and transfer materials to different destinations. The materials can either be recycled back to the cell surface, sent to late endosomes for degradation in the endolysosomal system, or transported to other destinations inside the cell [57]. Both the secretory and endocytic pathways cooperate in the delivery of molecules needed for essential biological processes within the cell, the secretion of molecules outside the cell, and the uptake of materials from the extracellular space; together, they enable internal homeostasis and communication with the extracellular environment.

The movement of materials via transport vesicles is tightly controlled by regulatory proteins that facilitate vesicle formation, cargo selection, and vesicle movement to ensure accurate packaging and delivery of cargo to the correct location. These regulatory components are specialized for different types of vesicles to enable appropriate spatiotemporal regulation of vesicle traffic between compartments and coordinate essential cellular processes [58]. For instance, in the ER‐Golgi network, the anterograde transport of newly synthesized lipids and proteins from the ER to the Golgi apparatus is facilitated by vesicles coated with the COPII protein complex [56], while the retrograde transport of escaped ER‐resident proteins and membrane components from the Golgi back to the ER is managed by vesicles coated with COPI proteins [59].

Central to vesicular trafficking is also the endolysosomal system, which orchestrates the degradation of internal cellular waste and materials external to the cell. In this system, clathrin‐coated vesicles internalize cargo, merging with early endosomes that are transported with the motor protein dynein along microtubules, and mature into acidic late endosomes that acquire hydrolytic enzymes from the Golgi apparatus. The process is completed when the late, mature endosomes fuse with lysosomes, where contents are fully digested and recycled [60]. Autophagy is a lysosome‐dependent degradation pathway that utilizes a distinct type of vesicles, the autophagosomes, to deliver targeted cellular components for recycling. Autophagosomes encapsulate damaged organelles and protein aggregates before fusing with lysosomes to form autolysosomes, where the breakdown of cargo occurs [61]. Crucial regulators of vesicular trafficking are Rab proteins, small GTPases that direct vesicles to specific cellular compartments. Different Rab proteins often correspond to specific stages of vesicular transport pathways and are considered markers of distinct vesicular transport pathways and compartments [58].

### Intercompartmental contact sites

In addition to the classic vesicular trafficking process, intracellular compartments can transfer materials directly through contact sites. The best studied are membrane contact sites (MCSs), found in specialized regions between two organelle membranes that are closely positioned (~30 nm apart) but remain unfused [62]. Instead, they are physically connected by tethering multiprotein complexes that bridge the narrow gap and coordinate the transfer of molecules between the two organelles to enable specific activities [63, 64]. The primary role of MCSs is to mediate the nonvesicular exchange of metabolites, but they are also fundamental for regulating organelle dynamics [65]. MCSs are most abundant across the extensive ER network, and elicit close and dynamic communication with most cellular components, including mitochondria, endosomes, the Golgi apparatus, lipid droplets, and the plasma membrane [1, 27, 64, 66, 67, 68, 69], but have been discovered connecting nearly all membranous organelles [70].

The best studied MCSs are those connecting the ER and mitochondria, known as mitochondria‐ER contact sites (MERCs) or mitochondria‐associated membranes (MAMs). Essential proteins in MERCs are inositol triphosphate receptor (IP3R), glucose‐regulated protein 75 (GRP75), and voltage‐dependent anion channel (VDAC). Together, they form the IP3R‐GRP75‐VDAC complex, which further cooperates with proteins such as mitofusin (MFN), vesicle‐associated membrane protein‐associated protein B/C (VAPB), or mitochondrial calcium uniporter (MCU) to carry out specialized functions [68, 71, 72, 73, 74] like mitochondrial dynamics, calcium signaling, lipid exchange, autophagy, the stress response, and apoptosis [42, 75, 76, 77, 78, 79, 80].

Studies on MCSs connecting the ER with the Golgi mainly focused on the ER‐TGN junction, where VAPs (VAMP‐associated proteins) act as key tethers [81]. These MCSs have been shown to facilitate the nonvesicular transfer of lipids, calcium, and other molecules necessary for coordinating regulatory crosstalk between the ER and Golgi [82, 83, 84].

MCSs between the ER and components of the endolysosomal pathway were found to regulate key processes such as cholesterol transfer, endosome fission, calcium exchange, and EGFR signaling [27, 85]. In particular, the crosstalk between the ER and endosomes through MCSs regulates STARD3‐mediated cholesterol transfer [86], attenuates EGFR signaling via the EGFR‐PTP1B interaction [87], and drives endosome fission through the spastin‐IST1 interaction [88]. Additionally, ER‐lysosome MCSs involve IP3R channels to facilitate calcium delivery from the ER to the lysosome [89].

The MCSs connecting mitochondria and lysosomes play a crucial role in mediating crosstalk between these organelles, impacting their dynamics, calcium exchange, and lipid metabolism [90, 91]. These interactions are regulated by the cooperation of various MCS proteins. For instance, the Rab7 (RAB7A) and Drp1 (DNM1L) GTPases regulate mitochondria and lysosome dynamics [92, 93]. The transient receptor potential mucolipin 1 (TRPML1, a lysosomal calcium efflux channel) along with VDAC1 and MCU regulate calcium signaling [94], while NPC2 and NPC1, STARD3, and ORP1L regulate lipid and cholesterol transfer [95]. There is intense interest in elucidating additional aspects of mitochondria‐lysosome MCSs and their implications in various diseases [96].

Recent advances in optical imaging have revealed dynamic interactions between various organelles and the cytoskeleton, providing new insights into their intricate communication [97]. These techniques have also highlighted physical connections between organelles and biomolecular condensates [5, 47, 49, 98] and have revealed specialized contact sites connecting the ER membrane with SGs and P‐bodies, two types of membraneless ribonucleoprotein (RNP) condensates. These discoveries indicate that ER contact sites are involved in regulating the biogenesis and disassembly of membraneless organelles [99]. This role of the ER in the regulation of SG dynamics has been supported by other studies [100, 101]. The interactions between the ER and biomolecular condensates can regulate condensate dynamics, mRNA localization, localized translation, and protein secretion [102]. For example, interactions between lysosomes and RNP granules, facilitated by the ANXA11 protein, permitted the lysosome‐mediated long‐distance transport of RNA along neuronal microtubules [103, 104].

The composition and functions of many contact sites, such as those involving the ER and mitochondria, have been reported. However, these multiprotein junctions connect nearly every cellular compartment, regulating a vast and complex communication network [70]. While much is known, the precise roles and regulatory mechanisms of many contact sites are still being actively investigated, and ongoing research utilizing novel technologies continues to provide new insights.

### Flow of regulatory signaling molecules

In addition to vesicular transport and contact sites, the effective cellular communication and integrated activities of intracellular compartments are tightly coordinated by signaling molecules, including transcription factors, enzymes, hormones, growth factors, ions, gases, and other small molecules that cross the membranes and/or cytosol via active transport or diffusion [9]. Among them, large macromolecules, small molecules, and ions are actively transported or passively diffuse through channels in the nuclear pore complexes (NPCs) to facilitate communication between the cytosol and the nucleus [105, 106, 107]. Meanwhile, small, water‐soluble second messenger molecules such as cAMP and cGMP are able to freely diffuse through concentration gradients in the cytosol to reach their target proteins located at the organelle surface [108]. When activated by cellular stimuli, these molecules rapidly initiate intracellular signaling cascades that coordinate compartmental function in response to cellular requirements. This coordination allows for precise control of cellular activities, including regulating gene expression and adjusting the cell metabolism to ensure proper organelle function, respond to stimuli, and maintain cellular homeostasis. The interaction between compartmentalization and signaling is crucial for metabolic flexibility, cellular adaptation, strength of the response, and optimal cellular function. Examples of this interplay include mitochondrial calcium signaling, lysosomal autophagy signaling, and ER stress signaling, such as the UPR [16].

## Morphological and functional changes of key cell compartments in senescence

**Fig. 3:**
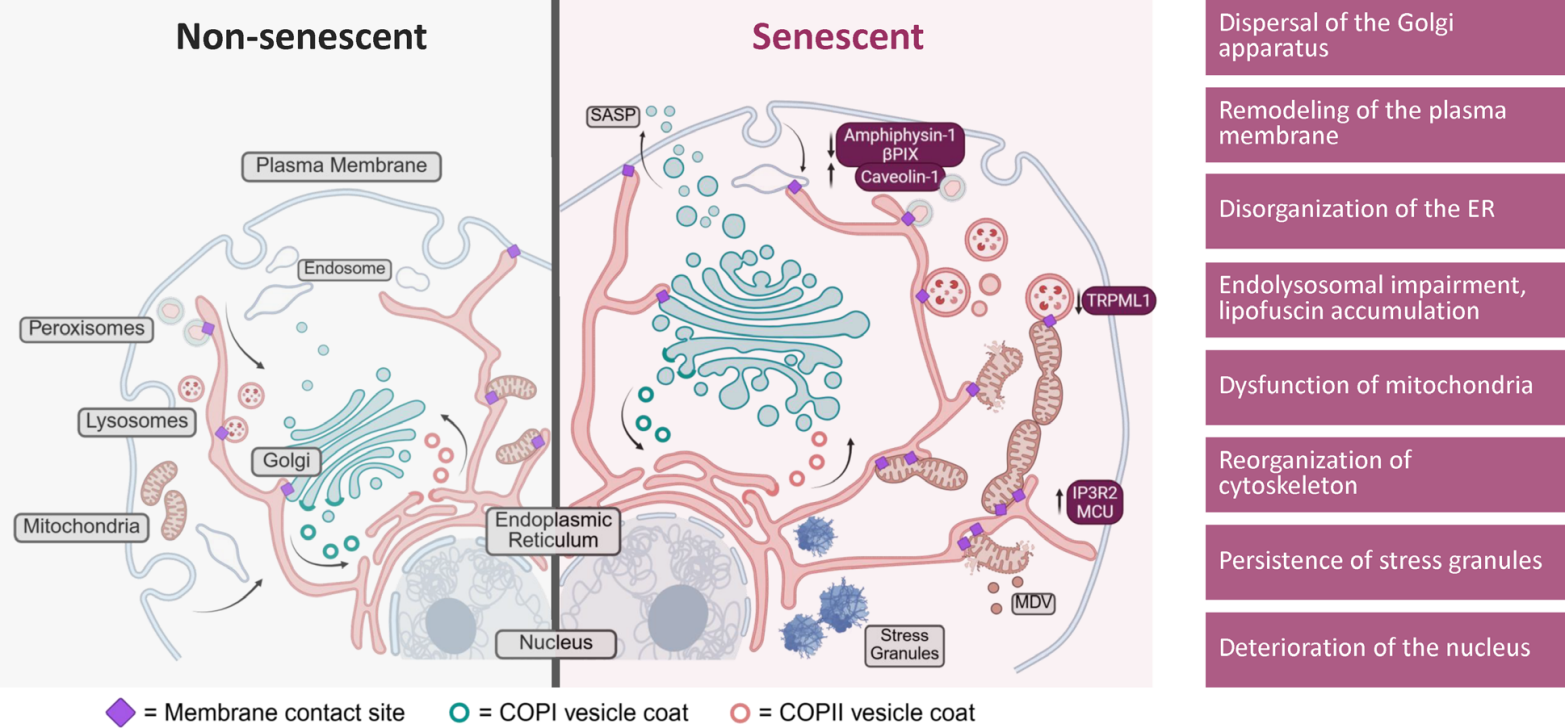
Senescence‐associated changes in structure and function of cell compartments, leading to altered communication. Schematic representation of nonsenescent and senescent cells highlighting senescence‐associated alterations in key cellular compartments. Senescent cells undergo remodeling of the plasma membrane, nuclear deterioration, disorganization of the ER, dispersal of the Golgi apparatus, impairment of the endolysosomal compartment with lipofuscin accumulation, dysfunctional mitochondria, reorganization of the cytoskeleton, and persistence of stress granules. These changes disrupt communication across compartments and contribute to impaired cellular homeostasis. Image was created using BioRender.

The architecture and function of intracellular components are essential for the execution of cellular processes that maintain cell homeostasis. Abnormalities in any of the components can impact the entire system leading to cellular impairment. While senescent cells have not endured damage severe enough to result in death, they display profound phenotypic changes, including changes in gene expression programs, an antiapoptotic status (whereby they remain alive despite persistent macromolecular lesions), alterations in organelle function, and acquisition of the SASP. These changes arise from complex and interconnected events that compromise the morphology and function of intracellular compartments (Fig. 3). Senescent cells frequently increase in size and alter their shape, which is typically accompanied by morphological changes in their internal compartments. These structural modifications can hinder the function of cell compartments and disrupt their intracellular interactions, ultimately changing the overall activity of the cell [109].

### Remodeling of the plasma membrane

The lipids in the plasma membrane are crucial for internal cell function, as they determine the distribution and function of membrane‐associated proteins, such as receptors and channels that control cell communication with the extracellular environment. During senescence, the plasma membrane undergoes metabolic changes in its lipid composition, such as increased levels of ceramide, a key component of sphingolipid metabolism, leading to the accumulation of ceramide‐rich rafts [110]. The remodeling of the plasma membrane composition further disrupts key cellular functions, such as signaling, membrane trafficking, and interaction with intracellular components. Such changes affect the cell's response to external stimuli and compromise the regulation of material transfer through secretory and endocytic pathways, contributing to the development of the SASP and reducing endocytosis in senescence [111, 112]. These changes can lead to cellular dysfunction and were linked to increased susceptibility to age‐related conditions such as neuronal dysfunction and chronic inflammation [113, 114].

### Deterioration of the nucleus

Senescent cells display an enlarged and irregularly shaped nucleus with invaginations and disruption of the nuclear envelope [115, 116, 117]. They also undergo extensive reorganization of the structure and composition of the chromatin, including the formation of senescence‐associated heterochromatin foci and a loss of heterochromatin leading to aberrant gene expression patterns that compromise cell function [118, 119]. Degeneration of the nuclear envelope in senescence is associated with the loss of key proteins that support the function of the nuclear pore complex (NPC), such as the transcription factor Sp1, nucleoporins, importins, exportins, and lamins. Since the NPC serves as a gateway for molecules to pass between the nucleus and cytoplasm, its disruption leads to the breakdown of compartmentalization, allowing leakage of materials, impairing nucleocytoplasmic transport, and dysregulating signaling pathways [120, 121]. For instance, misplaced chromatin fragments in the cytosol lead to the activation of the cGAS‐STING signaling pathway and the initiation of the SASP in senescence [122, 123, 124].

### Disorganization of the ER

Remodeling of the ER membrane architecture and modification of surface area in senescent cells are associated with compromised ER functions and altered signaling, including reduced UPR signaling in response to cellular stress [125, 126]. Given that a major role of ER is to maintain cellular proteostasis, a decline in ER function during senescence results in the accumulation of misfolded or unfolded proteins in the ER. This accumulation triggers persistent and unresolved ER stress signaling and impaired UPR activation, increasing the production of inflammatory factors in senescence [127]. Moreover, the changes in membrane structure and compositional features of the ER network were shown to involve the UPR sensor ATF6A, adding more complexity in the relationships between the UPR pathway and ER morphology [128, 129]. In addition, the structural changes and malfunction of the ER in senescent cells disrupt extensive interactions of the ER with other organelles, leading to impaired regulation of various processes, including autophagy and metabolism of calcium and lipids [1].

### Dispersal of the Golgi apparatus

The stacked arrangement of the Golgi apparatus cisternae is maintained by the cytoskeleton, GRASPs (Golgi Reassembly and Stacking Proteins), and golgins, along with biomolecular condensates, and plays a vital role in the proper processing, sorting, packaging, and distribution of proteins and lipids to their intended destinations [130, 131, 132]. Senescence is associated with alterations of the Golgi structure, including its dispersal, enlargement, and extension [133, 134, 135]. In one report, changes in Golgi structure during senescence were associated with increased levels and translocation of the signaling protein G subunit γ11 from the plasma membrane to the Golgi [134]. In another study, senescence‐associated Golgi changes were linked to reduced levels of the vacuolar ATPase ATP6V0A2, involved in protein glycosylation and organelle acidification; decreased ATP6V0A2 levels in turn disrupted pH balance and impaired Golgi trafficking and glycosylation, leading to Golgi stress and cellular senescence [135]. Recent studies uncovered that dysfunction of the Golgi contributes to the decline in function and regenerative capacity observed in aging mesenchymal stem cells [136], while age‐related zinc deficiency compromises Golgi stacking, impairing Golgi‐mediated glycosylation, vesicle transport, microtubule assembly, and protein localization [137].

Golgi fragmentation is also an early occurrence in age‐related neurodegenerative diseases such as AD and PD, where it is associated with disruptions in intracellular trafficking, cytoskeletal changes, and the accumulation of protein aggregates [132, 138, 139, 140]. Golgi dysfunction disrupted trafficking and processing of amyloid precursor protein (APP), resulting in increased production of amyloid beta peptides (Aβ) in an AD mouse model [141]. In short, Golgi malfunction during senescence can disrupt intracellular vesicular trafficking and cellular processes, leading to a disturbance in cellular homeostasis. A comprehensive understanding of how Golgi fragmentation contributes to dysfunctional cellular trafficking in cell senescence and neurodegenerative disease will require dedicated future studies that account for the heterogeneity of senescence across cell types, stimuli, damage levels, and tissue environments.

### Endolysosomal impairment

One of the key traits of senescent cells is the presence of increased numbers of enlarged dysfunctional lysosomes with damaged membranes, reduced acidity, and diminished proteolytic activity. This lysosomal impairment is reflected in the accumulation of undigested auto‐fluorescent lipofuscin and increased function of a β‐galactosidase active at pH 6, which are commonly employed markers of the senescent state [142]. Because the main function of lysosomes is to degrade and recycle cellular waste, lysosomal malfunction leads to the harmful accumulation of undigested material, which impairs critical processes like autophagy, calcium signaling, and endocytic and exocytic trafficking, disrupting the function of other organelles [143, 144]. A notable example is a feedback loop between lysosomes and mitochondria (the lysosome‐mitochondria axis) whereby failure of senescent lysosomes to clear damaged mitochondria through mitophagy leads to an accumulation of dysfunctional mitochondria, elevating ROS and oxidative stress, which further impairs lysosomal function and perpetuates cycle arrest [144, 145].

### Dysfunction of mitochondria

Mitochondrial dysfunction, characterized by reduced mitochondrial respiratory capacity and membrane potential, is a key hallmark of senescent cells [146]. Morphological changes of mitochondria in senescence include increases in mass, elongation, and hyperfusion of the mitochondrial network. Mitochondria form an interconnected network in the cytoplasm, constantly moving along cytoskeletal microtubules and altering their shape through fusion and fission. This dynamic mitochondrial structure, together with cellular quality control processes like mitophagy, is fundamentally altered in senescence, leading to the accumulation of dysfunctional mitochondria. These modifications both trigger and result from the dysfunction of organelles and the disruption of intracellular homeostasis in senescence [109, 147, 148, 149].

Senescence also disrupts the extensive interactions that mitochondria maintain with other cellular compartments, including the nucleus, ER, lysosomes, Golgi, and peroxisomes. In turn, this failure of coordination diminishes both mitochondrial and overall cellular homeostasis, promoting chronic dysfunction in the cell. As such, senescent cells exhibit impaired oxidative phosphorylation, reduced energy production, and increased oxidative stress; together, these changes lead to metabolic dysfunction and the secretion of pro‐inflammatory factors implicated in age‐related decline and disease [146, 148, 149, 150].

### Persistence of stress granules

Although biomolecular condensates have not been studied extensively in senescence, SGs have been examined. In healthy cells, SGs are typically dynamic, rapidly forming RNP aggregates that help cells adapt to acute stress conditions and disassemble once the stress is alleviated. However, the gradual accumulation of chronic stress and cellular damage during cellular senescence often leads to the formation of persistent and static SGs that fail to disassemble properly. The occurrence of aberrant SGs in senescence is influenced by malfunction of various cellular compartments and processes that govern their formation, composition, and disassembly. For instance, while the cytoskeleton and its associated motor proteins are crucial for SG formation and positioning [151, 152], senescent cells exhibit significant cytoskeletal rearrangements [153]. Similarly, autophagy is vital for clearing aberrant SGs [154], but senescent cells experience impaired autophagy and other internal degradation systems [155]. These age‐related cellular changes contribute to the abnormal assembly and persistence of SGs, making them an area of intense focus, particularly in age‐related neurodegeneration [156, 157, 158].

### Cytoskeleton reorganization

The cytoskeleton plays a crucial role in maintaining cell structure, shape, mechanical properties, and cellular transport. In senescent cells, variations in cell size, shape, and movement are associated with alterations in the architecture of the cytoskeleton. These changes include increased stability and acetylation of microtubules, modifications in actin filaments, and elevated levels of vimentin [109, 153, 159, 160]. The reorganization and stabilization of the cytoskeleton create a denser and more extensively crosslinked network of filaments that lead to changes in cell mechanical properties, such as enhanced cytoplasm viscosity during senescence [161, 162, 163]. This altered biophysical environment hinders the movement of motor proteins carrying vesicles and organelles like mitochondria and lysosomes, leading to disruptions in cellular processes like transport and recycling, and contributing to the dysfunction of senescent cells [159]. Age‐related increases in cytoplasmic viscosity and reductions in diffusiveness were reported in the neuronal cell body, but not in the axons, suggesting a compartmental specificity of this process [164]. While the exact mechanisms by which cytoskeletal modifications contribute to intracellular transport in cellular senescence are not fully understood, these findings highlight the significance of cytoplasmic mechanics in the impaired intracellular functions seen in senescence.

## Alteration of compartmental communication during senescence

### Disturbance of vesicular trafficking in senescence

Vesicular trafficking is a highly regulated, multi‐step process involving vesicle formation, transport, and fusion, coordinated by the precise collaborative action of various cell compartments and molecular signals [55]. Disrupting any step in this process can trigger cell dysfunction and contribute to senescence; conversely, cell senescence influences the expression levels of proteins implicated in the formation of transport vesicles and the assembly of cargo for different trafficking pathways (Table 1). In this regard, increasing evidence links dysregulated vesicular transport in senescent cells to age‐related pathologies like AD and PD [165, 166].

**Table 1:** Molecules implicated in intercompartmental communication, altered in senescence. Representative proteins and regulatory factors functioning in vesicular trafficking or interorganelle contact sites, whose abundance or activity is altered in senescent cells. Changes in these molecules contribute to impaired endocytic transport, dysregulated calcium mobilization, dysfunction of organelle communication, and overall loss of cell homeostasis.

Molecule	Change in senescence	Type of communication	Effect on cell function	References
Amphiphysin‐1	Decrease	Endocytic vesicular transport	Reduced clathrin‐mediated endocytosis	[112]
βPIX	Decrease	Endocytic vesicular transport	Reduced clathrin‐mediated endocytosis	[170]
Caveolin‐1	Increase	Endocytic vesicular transport	Dysfunctional caveolae	[174]
IP3R2	Increase	ER‐mitochondria MCSs	Increased calcium in mitochondria	[194, 195]
MCU	Increase	ER‐mitochondria MCSs	Increased calcium in mitochondria	[194]
VDAC1, MIC60, MTX1, SAMM50	Decrease	ER‐mitochondria MCSs	ER‐mitochondria contact sites dysfunction	[198]
TRPML1	Decrease	Lysosome‐mitochondria MCSs	Lysosomal dysfunction	[202]
ZEB1‐AS1	Increase	Lysosome‐mitochondria MCSs	Lysosomal dysfunction	[202]

Intracellular vesicular trafficking is centrally coordinated by the Golgi apparatus, which modifies, sorts, and packs proteins and lipids synthesized in the ER for distribution within the cell. In senescent cells, however, frequent distortion of the Golgi structure disturbs molecular processing and delivery of cargo to their destination compartments. This, in turn, compromises the performance of other organelles and attenuates cellular processes contributing to overall cellular dysfunction. One prominent example is the impairment of the endolysosomal and autophagy pathways in senescent cells; this process is frequently associated with the deformation of the Golgi system, which can hinder the sorting and vesicular delivery of digestive enzymes to lysosomes, compromising the breakdown and recycling of cellular components and leading to the accumulation of cellular waste [7, 167]. Recent studies have highlighted the role of Golgi‐resident GRASP proteins, crucial for the formation of Golgi structure and vesicle cargo sorting, in regulating Golgi‐lysosome communication, lysosomal enzyme trafficking, and autophagosome‐lysosome function [168, 169]. However, further work involving live‐cell imaging and proteome profiling is needed to explore more deeply the role of Golgi vesicular trafficking in endolysosomal and other communication networks in the context of senescence and aging.

Senescence‐associated changes in vesicular trafficking have been most extensively studied within endocytic pathways, which regulate the internalization and uptake of various substances from the plasma membrane. The levels of Amphiphysin 1, a key protein assisting in the formation of clathrin‐coated vesicles, were found to decrease in senescent human fibroblasts and in the lungs of old mice. This reduction led to the suppression of clathrin‐mediated endocytosis, a major route for forming vesicles that carry external materials into the cell [112, 170, 171]. At the same time, the abundance of Caveolin‐1 (CAV1), the main protein coating the endocytic vesicles caveolae, was reported to increase in senescence [172, 173]. While caveolae also mediate endocytosis, this increase has been linked to a dysfunctional state of the vesicles and impaired caveolae‐mediated endocytic pathway [174, 175]. In contrast, other studies have reported that reducing Caveolin‐1 levels induces mitochondrial dysfunction and premature senescence, suggesting that its function involves mechanisms beyond just protein levels [176]. Within the endosomal pathway, endocytosed cargoes are sorted in endosomes, with most material being sent to the lysosome for degradation, while specific cargoes are recycled via tubular carriers of the retromer protein complex back to the plasma membrane or to the TGN [177]. Interestingly, a functional retromer is crucial for lysosomal function, as evidenced in studies showing that a lack of the retromer coat subunit VPS35 can disrupt lysosomal structure and function, leading to impaired autophagy [178]. Similarly, the retromer subunit VPS29 is necessary for endolysosomal function in the aging brain of *Drosophila*, indicating a link between retromer dysfunction and aging [179]. Indeed, dysfunction of retromer‐mediated transport has been demonstrated in multiple age‐related neurodegenerative diseases, including AD and PD [177]. Further research leveraging proteomic and transcriptomic analyses is necessary to identify the components and interactions of the vesicular–tubular complex and fully understand its role in the cellular senescence program.

Recent studies have highlighted the role of the mitochondrial vesicular system in aging [180, 181, 182]. This system involves the formation of mitochondrial‐derived vesicles (MDVs) in response to mitochondrial stress. MDVs are important for mitochondria quality control and intracellular communication; they selectively package and transport damaged mitochondrial components and specific cargo to other organelles like peroxisomes, late endosomes, phagosomes, and lysosomes, to recycle damaged mitochondria and help cells adapt to stress. As mitophagy mechanisms are impaired with advancing age, MDVs were postulated to remove damaged mitochondrial material when the primary, bulk clearance method of mitophagy declines, but our understanding of MDVs is still quite limited.

### Disruption of compartmental contact sites in senescence

Alterations in the structure of intracellular compartments during cell senescence impact upon the function of their contact sites, impairing the exchange of information and molecules between compartments, and disrupting the coordination of cellular processes and overall cell function [1]. Given that contact sites have become increasingly recognized as crucial interorganellar communication platforms and regulators of cellular homeostasis, their dysfunction is increasingly associated with a range of diseases, including age‐related neurodegenerative conditions [4, 183, 184, 185].

The ER‐mitochondria contact sites, MERCs, are involved in the regulation of processes frequently dysregulated in senescent cells, such as calcium homeostasis, lipid biosynthesis and trafficking, ROS production, and autophagy; accordingly, these sites are being explored as potential therapeutic targets [186, 187, 188, 189, 190, 191, 192]. Some studies have shown that disruption in communication between the ER and mitochondria, caused by alterations in components of MERCs, leads to mitochondrial calcium overload, elevated ROS, and reduced mitochondrial membrane potential in senescence [193]. Specifically, during senescence, increased activity of the IP3R2 and higher levels of MCU channels (key regulators of the IP3R‐GRP75‐VDAC‐MCU complex in MERCs mediating calcium transfer from the ER to the mitochondria) lead to an accumulation of mitochondrial calcium and subsequent elevation in ROS production [194, 195, 196]. Additionally, senescent cells have been found to have increased MERCs [186, 195, 197]. Loss of the *Itpr2* gene (encoding IP3R2) in mice reduced the number of MERCs, decreased ER‐mitochondrial calcium flux, lowered ROS production, and mitigated age‐related characteristics. Furthermore, ectopic formation of MERCs impaired cell proliferation, raised levels of senescence markers like p16 and senescence‐associated‐β‐Galactosidase, and increased the expression of SASP components in mouse embryo fibroblasts [195]. Other studies in muscle of aging rats have shown decreased levels of specific proteins within the MERCs fraction, including VDAC1, MIC60, MTX1, and SAMM50, suggesting their role in age‐related MERC dysfunction [198]. Moreover, MERC dysfunction has been identified as a contributing factor to age‐related conditions such as AD, PD, and cardiovascular and metabolic diseases [71, 188]. Nonetheless, MERCs must be interpreted in light of their relative cellular mass, as increased tethering between the ER and mitochondria enhances Ca^2+^ transfer, boosting mitochondrial respiration but also increasing susceptibility to Ca^2+^ overload‐induced cell death [197].

The imbalance of calcium and lipid metabolism in senescent cells has prompted efforts to study the contact sites mediating crosstalk between lysosomes and mitochondria in age‐related diseases [90, 91, 93, 199, 200]. Recent research identified the lysosomal channel TRPML1, which plays a key role in regulating calcium exchange at lysosome‐mitochondrion contact sites, as influencing the function of both organelles [94]. Deficiency of TRPML1 was linked to mitochondrial dysfunction, increased ROS production, and impaired autophagy in natural killer (NK) cells [201]. Studies in old mice further demonstrated that the inhibition of TRPML1 by the long noncoding RNA *ZEB1‐AS1* contributes to lysosomal dysfunction, cardiomyocyte senescence, and accelerated heart aging [202]. Targeting TRPML1 function has been suggested as a potential therapeutic approach for AD [203], but further research is needed. While several other intercompartmental contact sites have been identified, their specific roles and regulation in senescence remain active areas of investigation.

### Defective flow of regulatory signaling molecules in senescence

The extensive structural and functional alterations to intracellular compartments in senescence impact the internal biophysical and chemical properties of the cell. These modifications have a significant influence on the dynamics and efficiency of transporting signaling molecules and cargo across cell membranes or the cytosol, ultimately affecting intracellular communication and homeostasis. Senescence‐related changes in the cytoskeleton correlate with a more rigid and less fluid cytoplasmic environment [161, 162], creating physical barriers that limit diffusion of signaling molecules, like cAMP and cGMP, and thereby influencing intracellular signaling pathways. Moreover, the altered spatial relationships between organelles in senescence hamper the diffusion of calcium ions, causing imbalances in calcium levels, and altering intracellular signaling processes regulated by calcium. Consequently, impaired signaling triggers a cascade of events that compromise cell homeostasis and the ability to respond to stress [1].

The nuclear deterioration and dysfunctional NPCs during senescence further compromise the active transport of macromolecules and the passive diffusion of ions and small molecules between the cytoplasm and nucleus. The disrupted translocation of regulatory cytoplasmic signals, like transcription factors, kinases, and metabolic enzymes, alters essential signaling pathways (e.g., MAPK/ERK and PI3K/AKT pathways) required for initiating specific transcriptional programs. These changes ultimately impair intracellular communication and reduce the cell's ability to respond to stimuli [120, 204, 205].

Modifications in plasma membrane composition observed in senescent cells, such as the increased ceramide‐rich lipid rafts, can restrict flow rates of signaling molecules or their receptors, disrupting normal signal transduction pathways [113]. These changes not only influence internal cellular signaling pathways but also affect communication of the cell with the external environment, including the release of SASP components and alteration of the ECM.

## Conclusions

Cellular compartments work together to regulate biological processes and maintain cell balance and responsiveness to stimuli. Changes in the structure of cell compartments during senescence can disrupt the function and communication between compartments; the ensuing lack of synchronization in cellular processes ultimately results in cell dysfunction, and has been implicated in age‐related neurodegeneration [185]. A deeper knowledge of these processes is crucial for understanding how the organelle ‘interactome’ is impacted during senescence and how it contributes to age‐associated diseases. Current innovations in super‐resolution microscopy, spatial proteomics, and other advanced technologies have enabled more precise visualization of connections between intracellular compartments and understanding the dynamics of their interactions [93, 97]. Together, these advancements are enabling an unprecedented exploration of how cell senescence affects communication across intracellular compartments and contributes to aging.

## Conflict of interest

The authors have no conflicts of interest to declare.

## Author contributions

KMM and EJW conceived and designed the review; KMM and MG wrote the paper; KMM, EJW, JL, and MG polished the paper; KMM, EJW, and JL prepared the figures.
